# Exploration of potential novel drug targets and biomarkers for small cell lung cancer by plasma proteome screening

**DOI:** 10.3389/fphar.2023.1266782

**Published:** 2023-09-06

**Authors:** Yijun Wu, Zhile Wang, Yuqi Yang, Chang Han, Li Wang, Kai Kang, Ailin Zhao

**Affiliations:** ^1^ Cancer Center, West China Hospital, Sichuan University, Chengdu, China; ^2^ Department of Thoracic Surgery, West China Hospital, Sichuan University, Chengdu, China; ^3^ West China School of Medicine, Sichuan University, Chengdu, Sichuan, China; ^4^ Department of Hematology, West China Hospital, Sichuan University, Chengdu, China

**Keywords:** drug target, biomarker, small cell lung cancer, plasma proteome, Mendelian Randomization

## Abstract

**Background:** Small cell lung cancer (SCLC) is characterized by extreme invasiveness and lethality. There have been very few developments in its diagnosis and treatment over the past decades. It is urgently needed to explore potential novel biomarkers and drug targets for SCLC.

**Methods:** Two-sample Mendelian Randomization (MR) was performed to investigate causal associations between SCLC and plasma proteins using genome-wide association studies (GWAS) summary statistics of SCLC from Transdisciplinary Research Into Cancer of the Lung Consortium (n_Case_ = 2,791 vs. n_Control_ = 20,580), and was validated in another cohort (n_Case_ = 2,664 vs. n_Control_ = 21,444). 734 plasma proteins and their genetic instruments of cis-acting protein quantitative trait loci (pQTL) were used, whereas external plasma proteome data was retrieved from deCODE database. Bidirectional MR, Steiger filtering and phenotype scanning were applied to further verify the associations.

**Results:** Seven significant (*p* < 6.81 × 10^−5^) plasma protein-SCLC pairs were identified by MR analysis, including ACP5 (OR = 0.76, 95% CI: 0.67–0.86), CPB2 (OR = 0.90, 95% CI: 0.86–0.95), GSTM3 (OR = 0.45, 95% CI: 0.33–0.63), SHMT1 (OR = 0.74, 95% CI: 0.64–0.86), CTSB (OR = 0.79, 95% CI: 0.71–0.88), NTNG1 (OR = 0.81, 95% CI: 0.74–0.90) and FAM171B (OR = 1.40, 95% CI: 1.21–1.62). The external validation confirmed that CPB2, GSTM3 and NTNG1 had protective effects against SCLC, while FAM171B increased SCLC risk. However, the reverse causality analysis revealed that SCLC caused significant changes in plasma levels of most of these proteins, including decreases of ACP5, CPB2, GSTM3 and NTNG1, and the increase of FAM171B.

**Conclusion:** This integrative analysis firstly suggested the causal associations between SCLC and plasma proteins, and the identified several proteins may be promising novel drug targets or biomarkers for SCLC.

## Introduction

Small Cell Lung Carcinoma (SCLC) is a highly aggressive and malignant subtype of lung cancer, accounting for approximately 14% of all lung cancer cases, characterized by its desperate invasiveness and poor prognosis (Wang et al., 2023). Although initially responsive to chemotherapy and radiation therapy, the majority of SCLC patients would develop treatment resistance within a short period, resulting in a very low overall survival rate (Rudin et al., 2021). While the emergence of immunotherapy has provided a breakthrough in the treatment of SCLC, its clinical efficacy remains unsatisfactory, with most patients experiencing relapse in the short term (Kang et al., 2023). The development of novel drugs for SCLC faces multiple challenges, including rapid growth and early metastasis, complex genetic characteristics, great tumor heterogeneity, and the issue of drug resistance, which make the search for effective drug targets and treatment strategies more difficult (Megyesfalvi et al., 2023; Zhang et al., 2023).

Mendelian randomization (MR) analysis, using single nucleotide polymorphism (SNP) from genome-wide association studies (GWAS) as genetic instruments, is a promising approach for drug target identification, which can effectively avoid confounding factors that exist in observational studies. Advances in genomics and proteomics have bolstered MR-based strategies, leading to potential therapeutic targets for diseases (Reay and Cairns, 2021). However, MR analysis integrating GWAS and protein quantitative trait loci (pQTL) data is lacking for SCLC, a method that can provide crucial insights into its early diagnosis and drug target discovery. Therefore, the objective of this study is to fill this gap, and first investigate plasma proteins as potential drug targets and biomarkers for SCLC by integrating large GWAS and pQTL data through MR analysis.

## Methods

### Plasma protein quantitative trait loci and GWAS data

**FIGURE 1:**
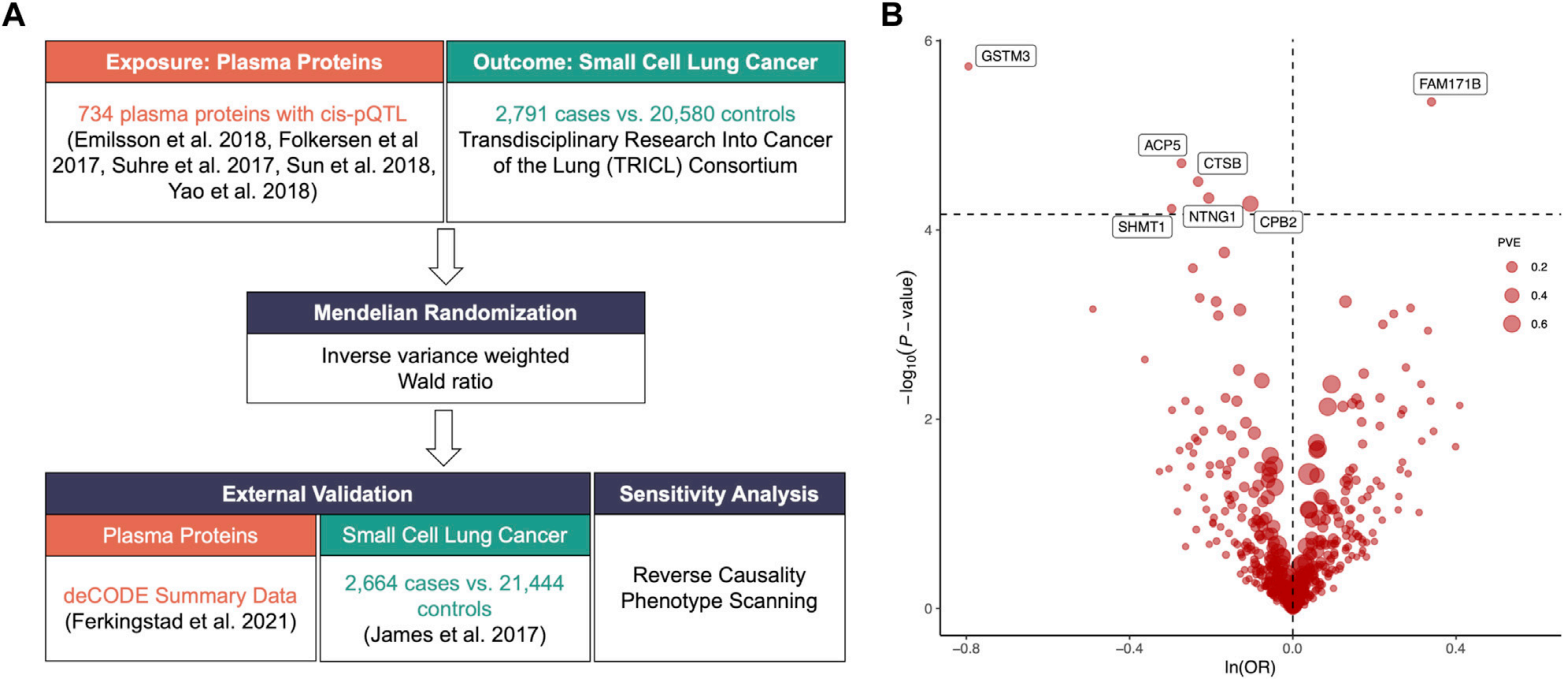
**(A)**. Flowchart of identifying causal plasma proteins for small cell lung cancer (SCLC) by Mendelian Randomization (MR). **(B)**. Volcano plot of the MR analysis for 734 plasma proteins on SCLC risk. OR: odds ratio, per standard deviation increase in plasma protein levels. Dashed horizontal line represented *p*-value = 6.81 × 10^−5^. PVE: proportion of variance explained.

We included plasm pQTL data from the integrated version (Zheng et al., 2020) of five previously reported GWAS datasets (Folkersen et al., 2017; Suhre et al., 2017; Emilsson et al., 2018; Sun et al., 2018; Yao et al., 2018) for primary analysis with 738 cis-pQTLs of 734 plasma proteins identified through the following criteria (Figure 1A; Supplementary Table S1): 1) being a cis-acting pQTL; 2) having significant genome-wide association (*p*-value < 5*10^−8^); 3) being situated beyond the boundaries of major histocompatibility complex region (chr6, 26–34 Mb); 4) demonstrating independent association with linkage disequilibrium clumping *r* ^2^ less than 0.001. The GWAS summary dataset for SCLC in the primary analysis was retrieved from Transdisciplinary Research Into Cancer of the Lung (TRICL) Consortium that enrolled 2,791 patients and 20,580 controls. Furthermore, external validation was performed using the plasma pQTL data of 4,907 proteins measured in 35,559 participants from Ferkingstad et al.’s study (Ferkingstad et al., 2021) and the SCLC GWAS summary dataset of 2,664 cases and 21,444 controls from James et al.’s study (McKay et al., 2017).

### Two-sample Mendelian Randomization analysis

Using the “TwoSampleMR” R package (https://github.com/MRCIEU/TwoSampleMR), plasma proteins were utilized as the exposure and SCLC as the outcome to conduct the primary Mendelian Randomization (MR) analysis. If there was only one pQTL available for a specific protein, we employed the Wald ratio method. For cases where two or more genetic instruments were accessible, we applied inverse variance weighted MR (MR-IVW) and performed subsequent heterogeneity analysis (Deng et al., 2022). In the primary analysis, we applied Bonferroni correction to account for multiple testing and set a threshold *p*-value of 0.05/734 (*p*-value < 6.81 × 10^−5^) to select potentially-valid causal proteins, which were then used to perform external validation at a threshold *p*-value of 0.05. During the external validation process, we also analyzed the results using the other two sets of genetic instruments of significant-variant and genome-wide significant SNPs based on the validation data.

### Sensitivity analysis

Significantly-valid genetic instruments for SCLC were identified from the TRICL Consortium dataset for the detection of possible reverse causality by bidirectional MR analysis. Five methods were used to estimate the causal effects, including MR-IVW, MR-Egger, weighted median, simple mode, and weighted mode. Meanwhile, we also performed Steiger filtering to verify the direction of associations between plasma proteins and SCLC. Phenoscanncer analysis (http://www.phenoscanner.medschl.cam.ac.uk) was used to identify the previously reported traits or diseases that shared the same SNPs with our analysis. *p*-value < 0.05 was considered statistically significant for these results.

## Results

The primary MR analysis identified seven significant plasma protein-SCLC pairs (*p*-value < 6.81 × 10^−5^; Figure 1B; and Table 1), including acid phosphatase 5 (ACP5), carboxypeptidase B2 (CPB2), glutathione S-transferase Mu 3 (GSTM3), serine hydroxy-methyltransferase 1 (SHMT1), Cathepsin B (CTSB), Netrin G1 (NTNG1), and Family With Sequence Similarity 171 Member B (FAM171B). In detail, higher levels of ACP5 (odds ratio, OR = 0.76, 95%CI: 0.67–0.86, *p* = 1.97 × 10^−5^), CPB2 (OR = 0.90, 95%CI: 0.86–0.95, *p* = 5.29 × 10^−5^), GSTM3 (OR = 0.45, 95%CI: 0.33–0.63, *p* = 1.86 × 10^−6^), SHMT1 (OR = 0.74, 95%CI: 0.64–0.86, *p* = 5.95 × 10^−5^), CTSB (OR = 0.79, 95%CI: 0.71–0.88, *p* = 3.07 × 10^−5^) and NTNG1 (OR = 0.81, 95%CI: 0.74–0.90, *p* = 4.60 × 10^−5^) decreased the risk of SCLC, while the elevated level of FAM171B (OR = 1.40, 95%CI: 1.21–1.62, *p* = 1.97 × 10^−5^) increased the risk of SCLC. Furthermore, there was no heterogeneity observed for the analyzed plasma proteins (Supplementary Table S2).

**TABLE 1:** Mendelian Randomization analysis for plasma proteins significantly with small cell lung cancer after Bonferroni correction.

Protein	UniProt ID	SNP	Effect allele	Or (95% CI)	*p*-value	PVE (%)	F statistics	Author
ACP5	A0A024R7F8; P13686	rs79061565	G	0.76 (0.67, 0.86)	1.97E-05	10.19	177.17	Sun et al
CPB2	A0A087WSY5; Q96IY4	rs3742264	T	0.90 (0.86, 0.95)	5.29E-05	62.70	454.41	Suhre et al
GSTM3	P21266	rs115929572	G	0.45 (0.33, 0.63)	1.86E-06	3.80	62.02	Emilsson et al
SHMT1	P34896	rs8067462	C	0.74 (0.64, 0.86)	5.95E-05	8.61	144	Emilsson et al
CTSB	Q5HYG5; A0A024R374; P07858; B4DMY4	rs1692819	A	0.79 (0.71, 0.88)	3.07E-05	13.56	240.14	Sun et al
NTNG1	Q5IEC3; Q5IEC8; Q9Y2I2; X5DNW2; B4DKF0	rs115668827	C	0.81 (0.74, 0.90)	4.60E-05	17.45	315.52	Sun et al
FAM171B	Q6P995	rs10931256	C	1.40 (1.21, 1.62)	4.42E-06	7.46	127.88	Sun et al

OR: odds ratio, per standard deviation increase in plasma protein levels. PVE: proportion of variance explained.

**FIGURE 2:**
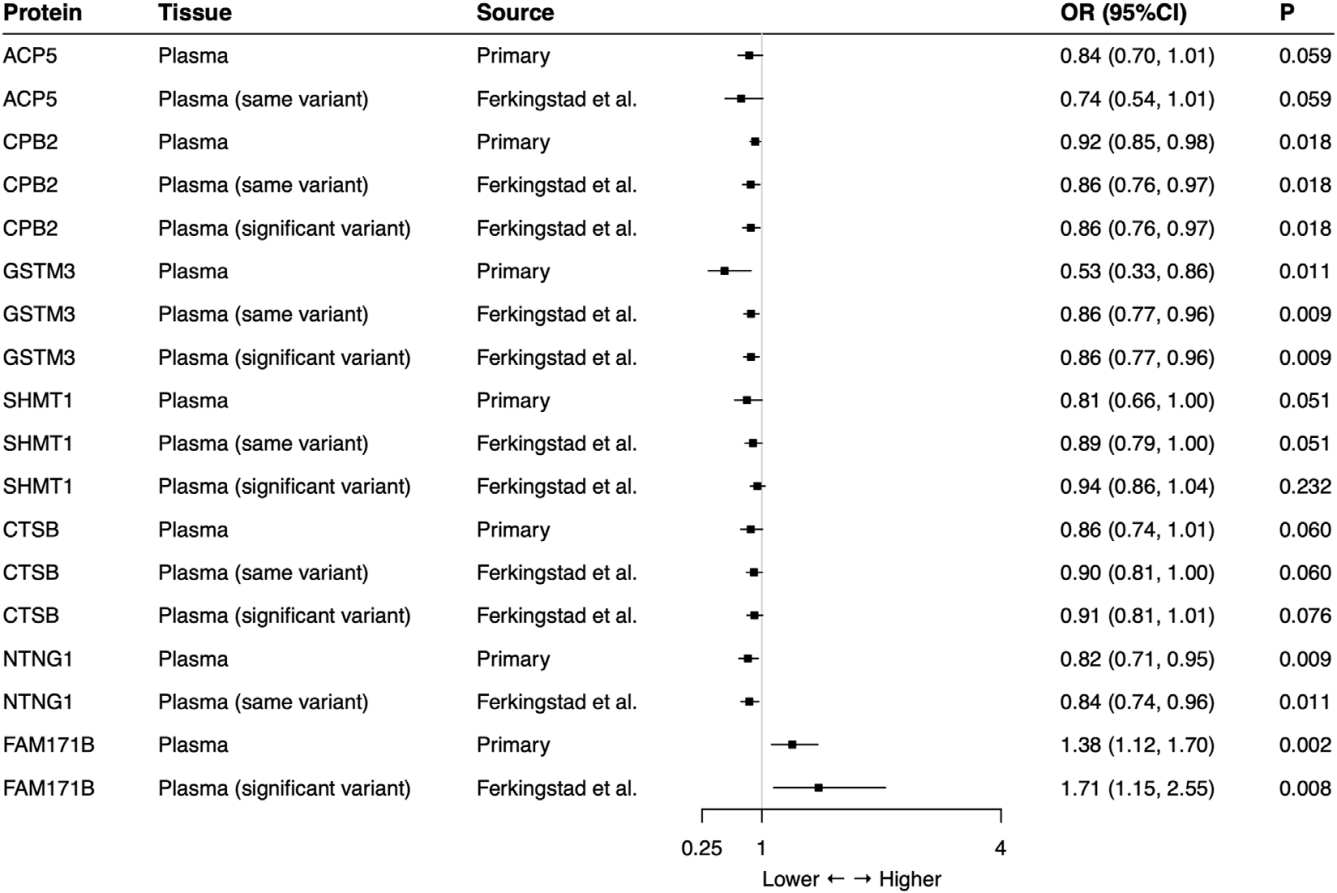
External validation of the causal relationship between small cell lung cancer and seven potential causal plasma proteins by Mendelian Randomization analysis. Same-variant strategy: using same SNP in the primary analysis but with its association in the external-validation data; significant-variant strategy: using the significant SNP in the external-validation data. ACP5 and NTNG1 had no available significant SNP and FAM171B had no same variant as the primary analysis in the external-validation data. OR: odds ratio, per standard deviation increase in plasma protein levels.

In the external validation, the same-variant and significant-variant strategies were used to further verify seven plasma proteins identified in the primary analysis, demonstrating that CPB2, GSTM3, NTNG1, and FAM171B also had significant associations with the risk of SCLC (Figure 2). For each protein, all used SNPs (primary, same-variant and significant-variant) showed consistent findings. For example, the elevated level of CPB2 always decreased the risk of SCLC when each of three types of SNP was used (primary: OR = 0.92, 95%CI: 0.85–0.98, *p* = 0.018; same variant: OR = 0.86, 95%CI: 0.76–0.97, *p* = 0.018; significant variant: OR = 0.86, 95%CI: 0.76–0.97, *p* = 0.018).

To further investigate the reverse causality, we next performed bidirectional MR analysis for seven potential plasma proteins in the primary analysis (genetic instruments shown in Supplementary Table S3), which suggests that SCLC caused significant changes in plasma levels of nearly all of them based on MR-IVW, except SHMT1 and CTSB, which had opposite changes between MR-IVW and other methods (Supplementary Figure S1). Overall, the occurrence of SCLC also caused changes in certain plasma proteins, including decreases of ACP5, CPB2, GSTM3, and NTNG1, and the increase of FAM171B. Finally, the phenotype scanning revealed that none of the identified proteins had reported associations with traits related to SCLC (Supplementary Table S4). Meanwhile, by Phenoscnner analysis, there were three SNPs in the primary analysis shared with other traits or diseases, including rs1692819 for CTSB, rs8067462 for SHMT1, and rs115668827 for NTNG1, which, however, had no explained relationship with SCLC (Supplementary Table S5).

## Discussion

As a highly invasive and rapidly progressing type of lung cancer, SCLC often presents a series of challenges, with the majority of patients being diagnosed at an advanced stage (Wang et al., 2022). Currently, there is a lack of effective drugs in clinical practice for SCLC treatment. Early diagnosis is crucial for treatment and prognosis, but SCLC patients typically lack obvious symptoms and biomarkers in their early stages, leading to difficulties in diagnosis. Often, by the time symptoms appear and further investigations are conducted, SCLC tumors have already been the advanced stage or spread, limiting opportunities for effective treatment (Megyesfalvi et al., 2023). Due to its rapid growth and lack of effective biomarkers, early diagnosis of SCLC is particularly challenging. In addition to the diagnostic challenges, treatment options for SCLC remain very limited, and there is a lack of effective drugs in clinical practice (Zhang et al., 2023). While some progress has been made over the past few decades, the progress in SCLC treatment has been relatively slow compared to non-small cell lung cancer. Immunotherapy has made significant progress in cancer treatment, but its applications and survival benefits in SCLC are still limited (Kang et al., 2023). Therefore, the difficulties in early diagnosis of SCLC and the lack of effective drugs are urgent issues to be addressed in clinical practice. It is critical to explore new diagnostic biomarkers and treatment drugs, aiming to provide more effective treatment options and improve the prognosis for SCLC patients.

In the present study, we firstly investigated the potential of plasma proteins as causal factors and biomarkers for SCLC incorporating large proteome datasets. By primary analysis and external validation, CPB2, GSTM3, NTNG1 and FAM171B were found to be associated with SCLC occurrence. However, according to the bidirectional MR analysis, ACP5, CPB2, GSTM3, NTNG1 and FMA171B obtained reliable changes when SCLC occurred, indicating their capabilities as biomarkers for SCLC. Noteworthily, only the increased FMA171B would increase the risk of SCLC, while others may play certain protective roles in SCLC occurrence, suggesting the individual importance of FAM171B. The protein-protein interaction analysis also demonstrated that FAM171B uniquely had no associations with other several proteins (Supplementary Figure S2).

FAM171B is an unidentified protein with its function yet to be clearly elucidated, and several studies have suggested its association with a diaphragmatic hernia and congenital heart disease (Li et al., 2015; Hauptman et al., 2018). FAM171B belongs to the Fam171b protein family, a group of secreted proteins highly selectively expressed in the brain (Hauptman et al., 2018). Therefore, our study, for the first time, identified FAM171B as a potential risk factor and biomarker for SCLC occurrence through large-scale plasma proteomics. However, its specific function and relationship with SCLC still require further investigation and exploration. Besides, other significantly protective protein molecules from SLC, whose potential functions and mechanisms remain unknown, have not been reported to be associated with SCLC., which may have potentials to become emerging drug targets and biomarkers for SCLC.

Several natural limitations are present in our study. First, the primary analysis was conducted based on plasma proteome datasets from multiple studies and measurement inconsistency might have caused bias, though most of the GWAS data sources were aptamer-based. Second, there is only cis-acting SNPs for all proteins, which limited further verifications. However, the SNPs for all proteins we finally identified had strong instruments with large values of F statistics (>10) and proportion of variance explained (PVE; >10%) except for GSTM3 with PVE = 3.8%. Finally, though we performed a series of validations and sensitivity analyses, the results we observed should be carefully explained, which are more suggestive rather than conclusive with regards to the causal identities and biomarkers of these proteins for SCLC. Thus, the future studies are required to validate these associations by developing a clinical cohort of SCLC patients and healthy controls: 1) choosing a group of SCLC patients and a group of healthy control subjects and ensuring that the two groups of samples are matched in terms of age, gender, and other potential confounding factors; 2) collecting plasma samples from each participant and ensuring that the collection method is standardized to minimize potential variability; 3) collecting tumor samples from SCLC patients to validate the expression levels of these potential molecules and their associations with plasma protein levels; 4) comparing the differences in plasma protein levels between SCLC group and the control group; 5) for molecules that do not express within SCLC tumors, potential origins should be further explored by investigating their expressions within normal human tissues of SCLC patients.

Overall, our study first investigates the causal associations between plasma proteins and SCLC by integrative analysis, revealing that circulating CPB2, GSTM3 and NTNG1 decreased SCLC risk, while FAM171B increased SCLC risk. In addition to them, ACP5 also exhibits an inverse causal relationship with SCLC, suggesting their potentials to be novel biomarkers for SCLC. Future studies are required to verify their specific roles in SCLC diagnosis and drug target development.

## Data Availability

The original contributions presented in the study are included in the article/Supplementary Material, further inquiries can be directed to the corresponding authors.

## Supplementary material

SUPPLEMENTARY FIGURE S1 Bidirectional Mendelian Randomization analysis for small cell lung cancer on plasma levels of seven potential causal proteins. OR: odds ratio, per standard deviation increase in plasma protein levels.

SUPPLEMENTARY FIGURE S2 Protein-protein interaction network among the suggestive causal proteins.
