# Supplementary figures and images for: Exploration of potential novel drug targets and biomarkers for small cell lung cancer by plasma proteome screening
Source: Front Pharmacol. 2023 Sep 6;14:1266782. doi: 10.3389/fphar.2023.1266782 (PMC10511877; doi:10.3389/fphar.2023.1266782)

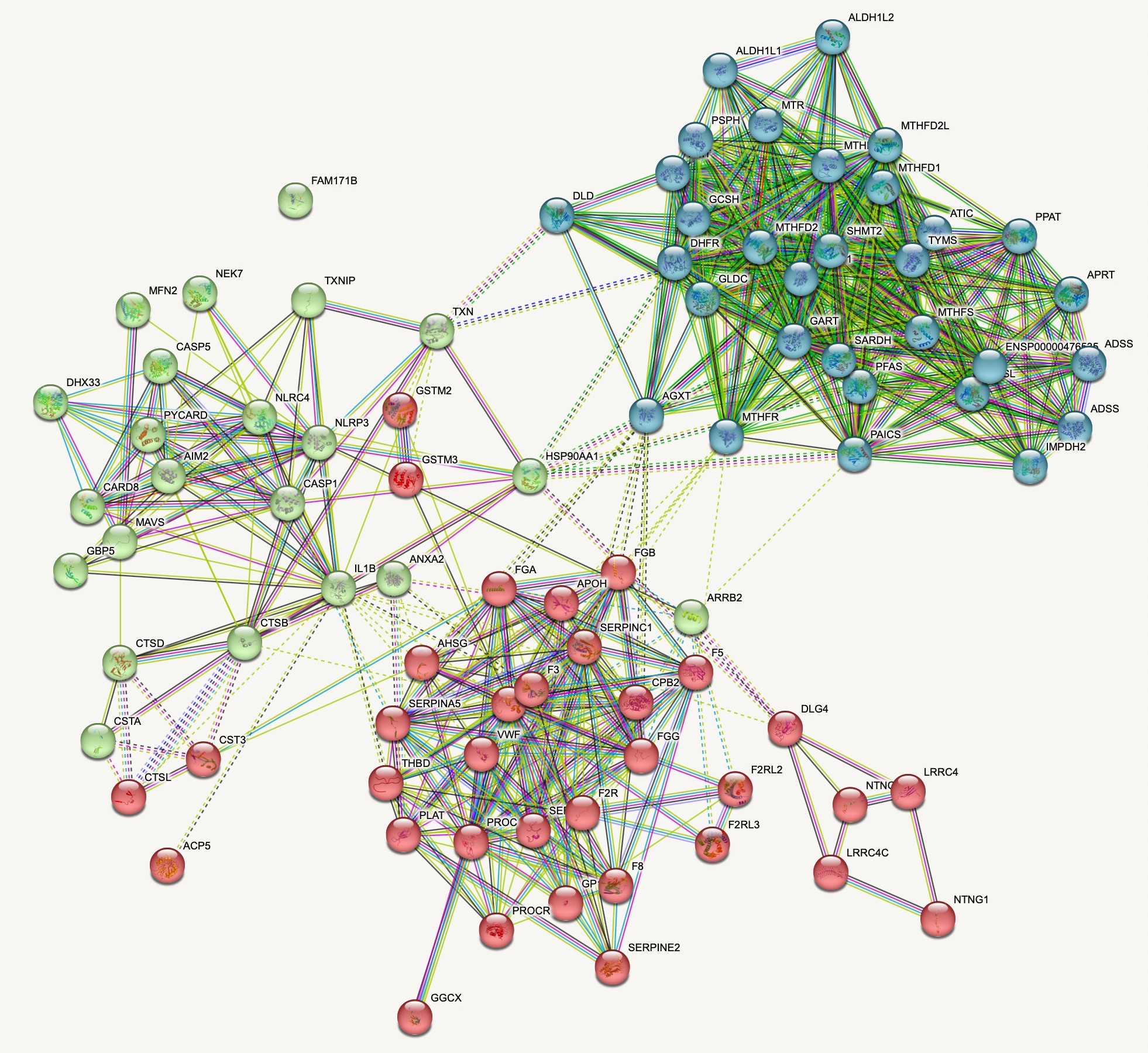

Supplement: Supplementary file 3 [file Image1.JPEG]

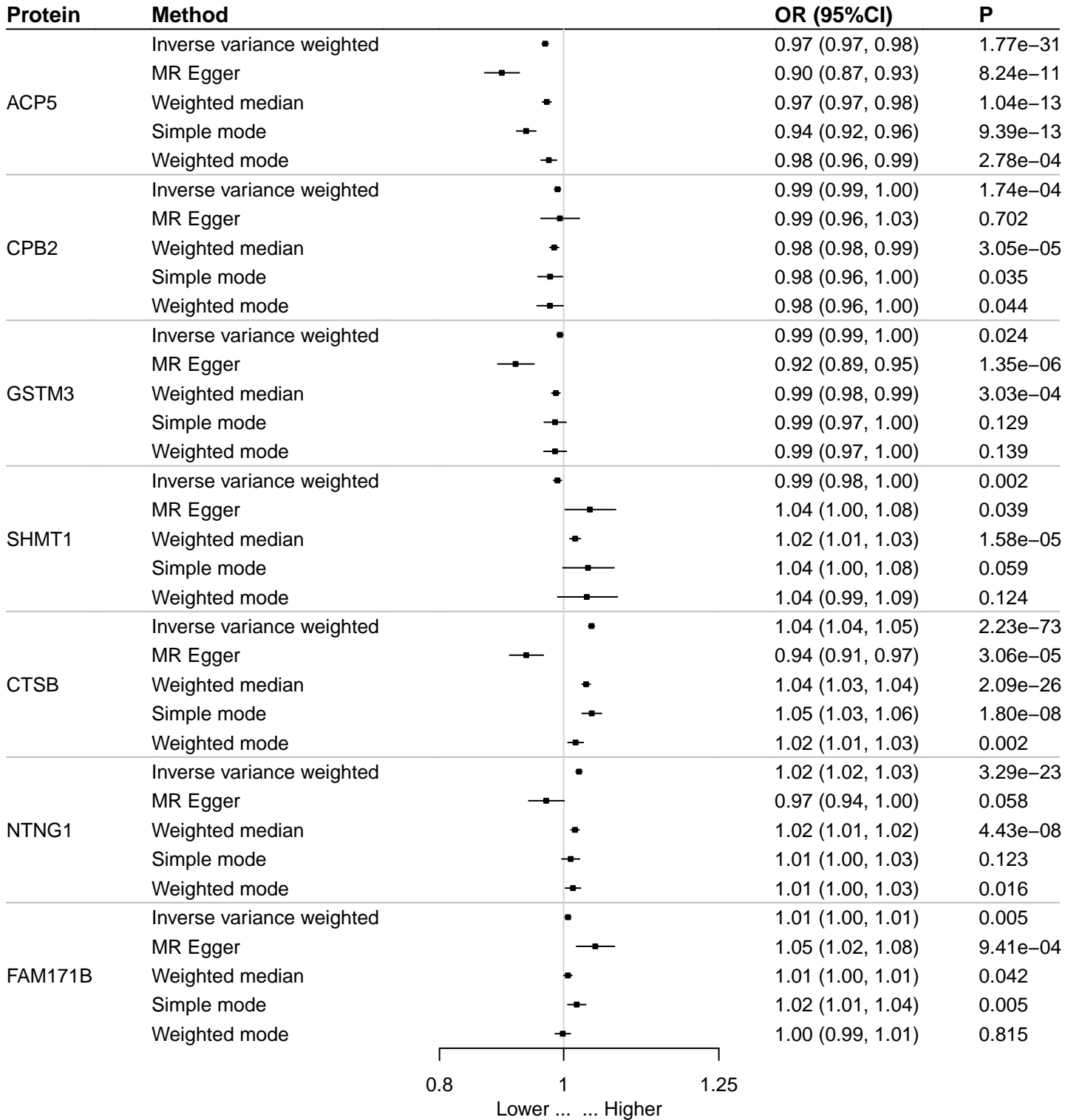

Supplement: Supplementary file 4 [file DataSheet1.PDF]
